# Supplementary material for: Vibrational properties of graphdiynes as 2D carbon materials beyond graphene
Source: Phys Chem Chem Phys. 2022 Apr 13;24(17):10524–36. doi: 10.1039/d2cp00980c (PMC9425158; doi:10.1039/d2cp00980c)
Supplement: CP-024-D2CP00980C-s001 [file CP-024-D2CP00980C-s001.pdf]

Electronic Supplementary Material (ESI) for Physical Chemistry Chemical Physics.
This journal is © the Owner Societies 2022

Supporting Informations

Vibrational properties of graphdiynes as 2D carbon materials beyond graphene

P. Serafini,[a] A. Milani,[a] M. Tommasini,[b] C. Castiglioni,[b] D.M. Proserpio,[c] C.E. Bottani,[a] C.S. Casari*[a]

[a.] *Department of Energy, Politecnico di Milano, Via Ponzio 23/3 20133 Milan, Italy.*
[b.] *Department of Chemistry, Materials and Chem. Eng. 'G.Natta' , Politecnico di Milano, Piazza Leonardo da Vinci 32 20133 Milan, Italy.*
[c.] *Dipartimento di Chimica, Università degli Studi di Milano, 20133 Milano, Italy*

Table S1. Sketch of the 2D-GDY and 2D-GZY structures analysed. For each structure the unit cell, space group point group symmetry (factor group at Γ point of BZ), number of polyyne chains belonging to the unit cell are illustrated. Energy contribution per carbon atom, referred to graphene (ΔE), bond alternation parameter (BLA) of the polyyne units are reported. (i) in the fourth column indicates that inversion symmetry centres are located on the the polyyne chains. The number and symmetry species of the ECC phonons are listed in the table.

structure	unit cell	point group<br><br>space group	number of chains	DE ($Kcal\ mol^{-1}$)	BLA (Å)	ECC modes
6-H<br><br>a -GDY	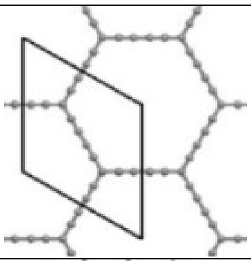	D_{6h}<br>p6mm	3 (i)	27.39	0.142	$1A_g+1E_{2g}$
6-L	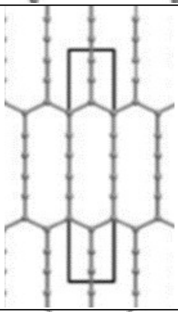	D_{2h}<br>c2mm	centred: 2<br>primitive:1 (i)	26.18	0.136	$1A_g$
6-R	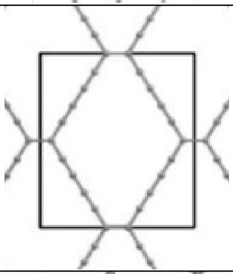	D_{2h}<br>c2mm	centred: 4<br>primitive: 2(i)	25.48	0.154	$1A_g+1B_{1g}$
6-HT[2]<br><br>b -GDY	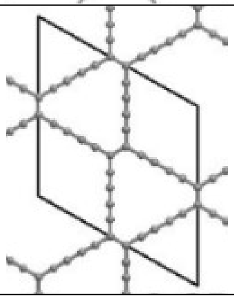	D_{6h}<br>p6mm	6	25.40	0.156	$1A_g+2E_{2g}$<br>(partial ECC character)

6-hT[2]<br><br>g -GDY		D_{6h}<br>p6mm	3(i)	21.13	0.167	$1A_g+1E_{2g}$
6-hL	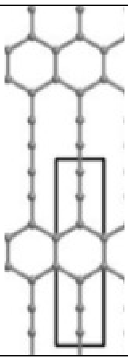	D_{2h}<br>p2mm	1(i)	20.59	0.138	$1A_g$
6-hL[2]	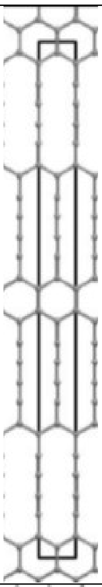	D_{2h}<br>c2mm	centred: 4<br>primitive: 2	23.11	0.136	$2A_g$<br>(partial ECC character)
6-hL[3]	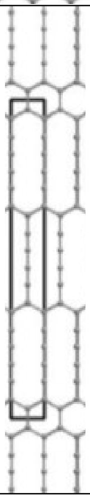	D_{2h}<br>p2mm	3<br>i(central chain only)	24.06	0.135	$3\ A_g$<br>(partial ECC character)

6-h^{2}L		D_{2h}/ c2mm	centred: 2<br>primitive: 1(i)	16.81	0.139	1A_g
6-h^{3}L		D_{2h}/ p2mm	1(i)	14.15	0.138	1 Ag

*Tabella S2. Computed wavenumbers (spectral region above 700 cm^{-1}), symmetry species, relative Raman activities and IR band intensities for the **q=0** Raman or IR active phonons of the GDY and GZY crystals.*

**2D- GDY structures**					
	Raman Spectrum			IR spectrum	
System	wavenumber (cm^{-1}) (Irrep)	Raman activity	ECC character	wavenumber (cm^{-1}) (Irrep)	IR intensity (km mol^{-1})
**6-H**	831 (E_{2g})	36		1300 (E_{1u})	1516
	1351 (A_{1g})	0		2219 (E_{1u})	365
	1498 (E_{2g})	337			
	2041 (E_{2g})	1000	ECC		
	2361 (A_{1g})	0.02	ECC		

**6-L**	1092 (B_{1g})	11		1182 (B_{3u})	14
	1107 (A_g)	35		2260 (B_{3u})	2
	1531 (A_g)	381			
	2118 (A_g)	1000	ECC		
**6-R**	1156 (A_g)	386		1036 (B_{2u})	302
	1404 (A_g)	749		1254 (B_{3u})	280
	1541 (B_{1g})	9		2241 (B_{3u})	267
	2193 (A_g)	1000	ECC	2269 (B_{2u})	20
	2202 (B_{1g})	57	ECC		
**6-HT^2**	1014 (E_{2g})	12		754 (E_{1u})	1
	1181 (A_{1g})	167		1185 (E_{1u})	1067
	1445 (E_{2g})	27		1452 (E_{1u})	482
	1448 (A_{1g})	621		2246 (E_{1u})	1501
	1532 (E_{2g})	109		2356 (E_{1u})	2
	2240 (E_{2g})	142	partial ECC character		
	2240 (A_{1g})	1000	ECC		
	2255 (E_{2g})	453	partial ECC character		
**6-hT^2**	790 (E_{2g})	1		893 (E_{1u})	14
	984 (A_{1g})	63		1429 (E_{1u})	1045
	1382 (E_{2g})	3		2269 (E_{1u})	480
	1491 (A_{1g})	235			
	1574 (E_{2g})	134			
	2277 (A_{1g})	1000	ECC		
	2337 (E_{2g})	344	ECC		
**2D-GZY structures**					
Raman Spectrum				IR spectrum	
System	wavenumber (cm^{-1}) (Irrep)	Raman activity	ECC character	wavenumber (cm^{-1}) (Irrep)	IR intensity (km mol^{-1})
**6-hL**	753 (B_{2g})	0		713 B_{1u}	1
	899 (A_g)	0		853(B_{3u})	331
	1342(B_{1g})	1000		1264(B_{2u})	1
	1482(A_g)	47		1435(B_{3u})	3085
	1567(A_g)	171		2255(B_{3u})	8719
	2248(A_g)	328	ECC		
**6-hL^2**	752 (B_{2g})	0		715 (B_{1u})	1
	761 (B_{2g})	0		987 (B_{3u})	0
	1000 (A_g)	5		1208 (B_{2u})	1
	1206 (B_{1g})	3		1339 (B_{3u})	99
	1296 (B_{1g})	3		1563 (B_{3u})	64
	1357 (A_g)	55		2255 (B_{3u})	584
	1531 (A_g)	223		2310 (B_{3u})	58
	1559 (A_g)	400			
	2205 (A_g)	1000	partial ECC character		

	2244 (A_g)	358	partial ECC character		
**6-hL^3**	753 (A_g)	0		705 (B_{3u})	938
	757 (B_{2g})	0		716 (B_{1u})	1
	765 (B_{2g})	0		753 (B_{1u})	0
	1040 (A_g)	8		1037 (B_{3u})	15583
	1177 (B_{1g})	633		1175 (B_{2u})	0
	1258 (B_{1g})	890		1183 (B_{2u})	1
	1305 (A_g)	31		1288 (B_{3u})	55108
	1501 (A_g)	84		1439 (B_{3u})	11410
	1549 (A_g)	359		1574 (B_{3u})	8304
	1563 (A_g)	42		2219 (B_{3u})	444
	2173 (A_g)	1000	ECC	2256 (B_{3u})	248804
	2245 (A_g)	107	CºC str	2292 (B_{3u})	88
	2313 (A_g)	15	ECC		
**6-h^{2}L**	728 (A_g)	1		735 (B_{1u})	1
	736 (B_{2g})	0		1288 (B_{3u})	83
	1228 (A_g)	16		1366 (B_{2u})	0
	1325 (B_{1g})	5		1567 (B_{3u})	271
	1443 (B_{1g})	71		2250 (B_{3u})	640
	1567 (A_g)	9			
	1577 (A_g)	856			
	2257 (A_g)	1000	ECC		
**6-h^{3}L**	737 (B_{2g})	0		720 (B_{1u})	1
	1089 (A_g)	2		1086 (B_{3u})	16
	1385 (B_{1g})	116		1359 (B_{2u})	0
	1458 (A_g)	13		1419 (B_{3u})	86
	1498 (B_{1g})	1000		1437 (B_{2u})	0
	1587 (A_g)	371		1614 (B_{3u})	192
	1612 (A_g)	81		2247 (B_{3u})	512
	2224 (A_g)	387	ECC		

Tabella S3. Sketches of the vibrational eigenvectors associated to the main active Raman and IR phonons discussed in the main text. Associated wavenumbers and irreducible representations are also reported for each phonon.

System	Wavenumber (cm^{-1})	Irrep
**6-R**		

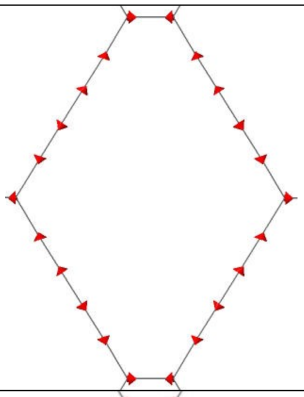	1156	A_g
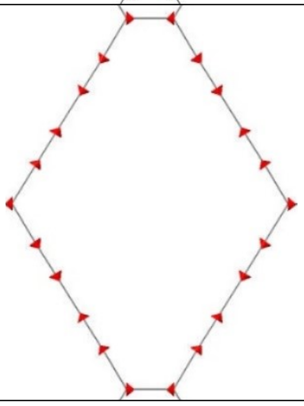	1404	A_g
**6-HT^2**		
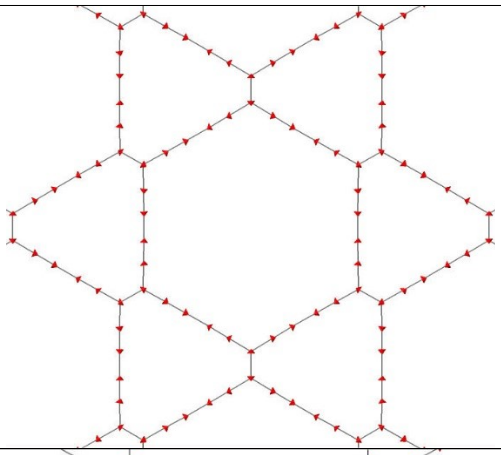	1181	A_{1g}
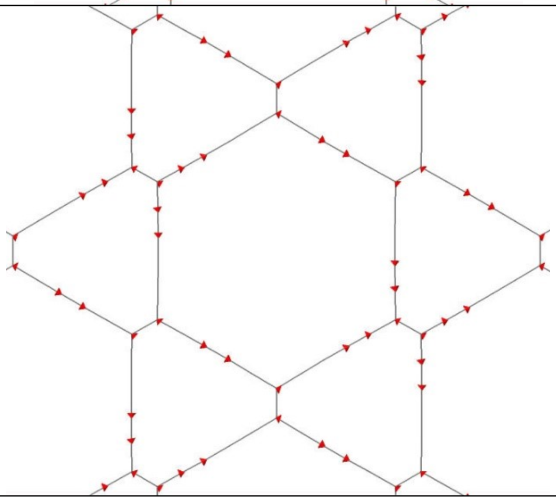	1185	E_{1u}

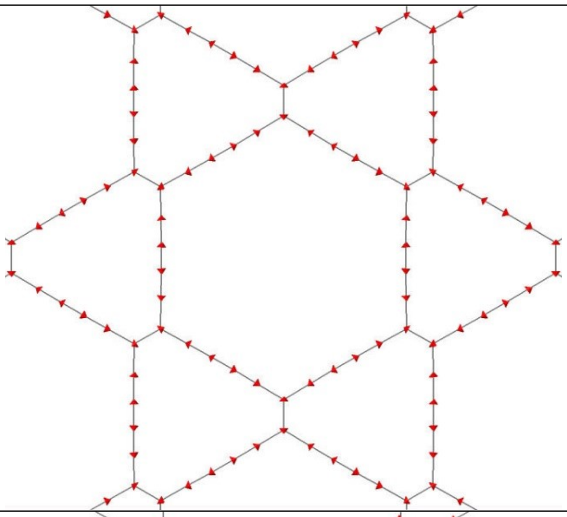	1448	A_{1g}
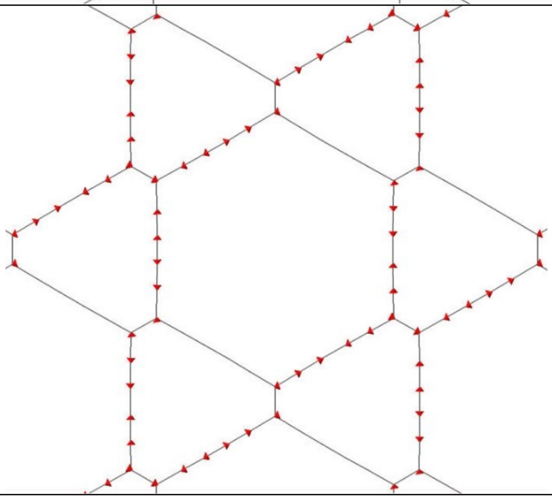	1452	E_{1u}
**6-hT²**		
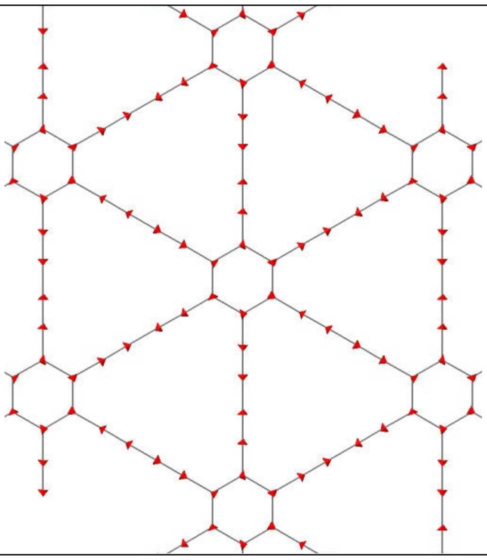	1382	E_{2g}

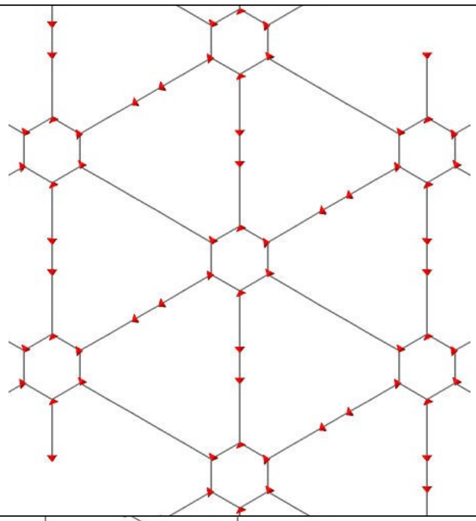	1429	E_{1u}
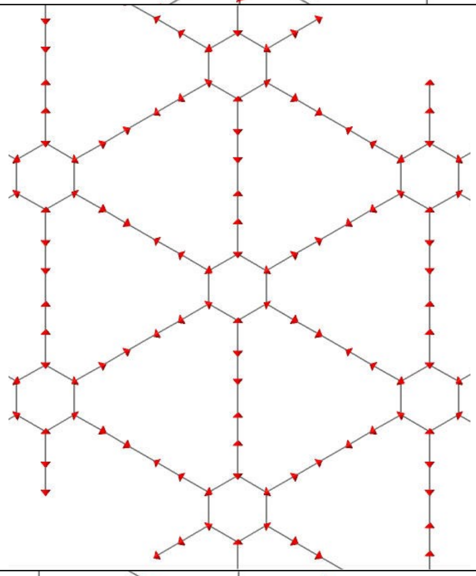	1491	A_{1g}
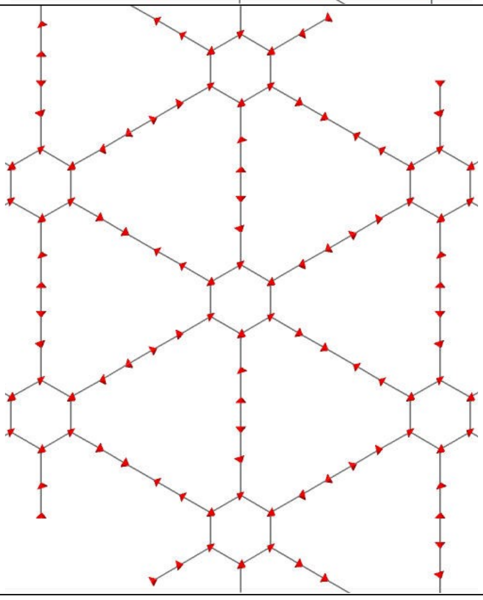	1574	E_{2g}
**6-H**		

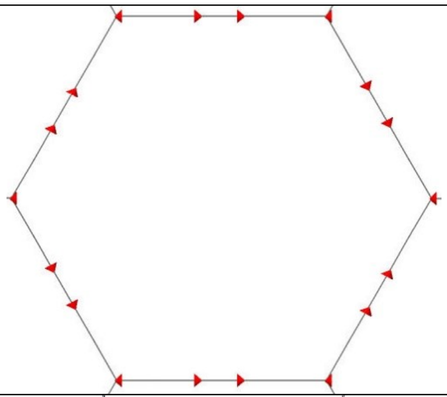	1300	E_{1u}
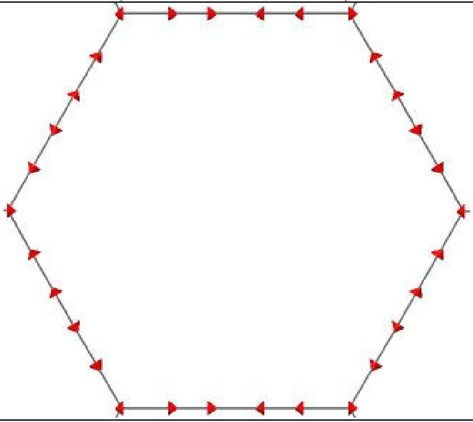	1498	E_{2g}
**6-L**		
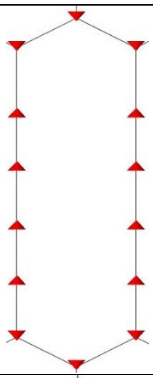	1182	B_{3u}
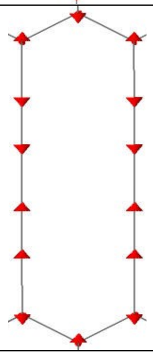	1531	A_g
